# Editorial: Acute Unilateral Vestibulopathy: clinical presentation, instrumental patterns, evolution and management

**DOI:** 10.3389/fneur.2023.1226601

**Published:** 2023-06-12

**Authors:** Salvatore Martellucci, Marco Mandalà, Augusto Pietro Casani, Dario Andres Yacovino, Andrea Castellucci

**Affiliations:** ^1^Otorhinolaryngology Unit, Hospital Santa Maria Goretti, Latina, Italy; ^2^University of Siena, Siena, Italy; ^3^Department of Surgical, Medical, Molecular and Critical Area Pathology, University of Pisa, Pisa, Italy; ^4^Department of Neurology, Dr. César Milstein Hospital, Buenos Aires, Argentina; ^5^Department of Surgery, ENT Unit, Azienda USL-IRCCS, Reggio Emilia, Italy

**Keywords:** Acute Unilateral Vestibulopathy, BPPV, hearing loss, video head impulse test (vHIT), head shaking test, medical therapies, vestibular compensation

Acute Unilateral Vestibulopathy: clinical presentation, instrumental patterns, evolution and management Acute Unilateral Vestibulopathy (AUV) represents severe, continuous, and long-lasting vertigo with sudden onset due to acute damage involving either the vestibular nerve or the labyrinthine end-organs. Although most subjects recover spontaneously, several patients develop residual disorders, such as chronic dizziness, disequilibrium, spatial disorientation, and limitations in daily activities. More than a century after the first description, Acute Unilateral Vestibulopathy (AUV) is a syndrome whose etiopathogenesis is still debated. It represents a challenging and intriguing pathology for clinicians from a diagnostic and therapeutic point of view, particularly for the aspects linked to its evolution and the development of vestibular compensation (1, 2).

As the title indicates, this Research Topic entitled “*Acute Unilateral Vestibulopathy: clinical presentation, instrumental patterns, evolution, and management*” covers some of the multiple aspects associated with losing the function of one of the two labyrinths in the light of the latest international guidelines (3, 4).

Two articles in this collection address the issue of the epidemiology and relative etiopathogenesis of AUV. Viberti et al. assessed the epidemiological features of AUV in three different districts in Italy, showing how the estimated incidence is higher than previously reported in the literature. During the COVID-19 pandemic, the epidemiology of several diseases has changed worldwide. The impact of COVID-19 vaccination on AUV development in the setting of a tertiary interdisciplinary neurotology center was retrospectively investigated by Schmid et al..

The video head impulse test (vHIT) is an irreplaceable tool for recognizing the different AUV patterns in an emergency department and following their evolution over time. Alfarghal et al. after a comprehensive literature review, examined the VOR gain assessed using the vHIT in acute vestibular syndromes and proposed a grading scale for the severity of VOR impairment of lateral semicircular canal similar to the current grading for hearing loss based on pure tone audiometry. The clinical implications of vHIT in patients suffering from BPPV secondary to idiopathic sudden sensorineural hearing loss were investigated by Liu et al. who demonstrated that in this subpopulation, the vestibular function and, in particular, the posterior semicircular canal appears to be impaired compared to what was found in patients affected by idiopathic BPPV. The vHIT devices currently in use usually record the track from only one eye. A newer vHIT device allowing a simultaneous record of binocular vHIT has been the subject of a cross-sectional, prospective study by Striteska, Chovanec et al.. The article provided normative values reflecting the conjugacy of eye movement responses to horizontal binocular vHIT in healthy participants.

Vestibular damage and hypofunction could be associated with sudden sensorineural hearing loss since cochleovestibular structures share the same vascularization and are in close anatomical proximity. A retrospective study was conducted by Castellucci et al. to evaluate the specific lesion patterns of vestibular damage in patients presenting with sudden sensorineural hearing loss with or without vertigo and assess the prognostic role of vestibular dysfunctions on hearing recovery, suggesting that vestibular evaluation in SSNHL can provide helpful information on hearing recovery and underlying etiologies.

The ability to compensate and the strategies with which this occurs represent the main question when diagnosing a vestibulopathy. Bedside and instrumental test batteries provide suggestions on each patient's ability to recover, but to date, there needs to be more data on the prognostic value of each test. In a prospective observational case-control study, Striteska, Valis et al. aimed to assess the ability of a head-shaking test (HST) to reflect vestibular compensation in patients after acute vestibular loss, showing how the intensity of nystagmus induced by HST decreased exponentially over time, declining to the value of the control group once vestibular compensation was satisfactory and sufficient for a patient's everyday life. In contrast, well-detectable head-shaking induced nystagmus in subjects with insufficient clinical recovery patients served as an objective indicator of poorly compensated unilateral vestibular loss. Vestibular compensation is strictly linked to the level of physical activity practiced after the acute event and reflects the patient's quality of life. The association between the level of physical activity and chronic dizziness was assessed by Van Laer et al. in a retrospective cohort study on 66 patients who underwent vestibular schwannoma resection.

The onset of an acute vestibular syndrome requires urgent pharmacological management due to the critical procession of symptoms accompanying the event, which therapeutic choices and intervention timing can positively or negatively influence. Viola et al. reviewed the pharmacological therapeutic option, correlating them to the differential and, as far as possible, to the etiological diagnosis.

Finally, this Research Topic includes Finally, this Research Topic includes the description of some clinical cases peculiar to rarity and iconography: the ossification of a posterior semicircular canal following an AUV and mimicking inferior vestibular neuritis was reported by Comacchio and Castellucci whereas three cases of cerebellitis in anti-Yo paraneoplastic syndrome were described and discussed by Kherallah et al..

The Editors hope that this Research Topic can represent a valuable contribution for all clinicians involved in otoneurology, particularly those involved in diagnosing and treating patients affected by acute vestibular syndromes.

## Author contributions

SM and ACast wrote the manuscript. All editors designed the Research Topic, reviewed the manuscript, and approved the submitted version. All authors contributed to the article and approved the submitted version.
